# Trends in Infectious Keratitis in Taiwan: An Update on Predisposing Factors, Microbiological and Antibiotic Susceptibility Patterns

**DOI:** 10.3390/diagnostics12092095

**Published:** 2022-08-29

**Authors:** Jin-Jhe Wang, Chien-Hsiung Lai, Chau-Yin Chen, Chia-Yen Liu, Meng-Hung Lin, Yao-Hsu Yang, Pei-Lun Wu

**Affiliations:** 1 Department of Ophthalmology, Chiayi Chang Gung Memorial Hospital, Chiayi 61363, Taiwan; 2 Department of Nursing, Chang Gung University of Science and Technology, Chiayi 61363, Taiwan; 3 School of Traditional Chinese Medicine, College of Medicine, Chang Gung University, Taoyuan 33302, Taiwan; 4 Health Information and Epidemiology Laboratory, Chiayi Chang Gung Memorial Hospital, Chiayi 61363, Taiwan; 5 Department of Traditional Chinese Medicine, Chang Gung Memorial Hospital, Chiayi 61363, Taiwan

**Keywords:** infectious keratitis, corneal infection, antibiotic susceptibility, antimicrobial resistance

## Abstract

Infectious keratitis (IK) represents a major cause of corneal blindness. This study aims to investigate the demographics, risk factors, microbiological characteristics and antibiotic susceptibility patterns of IK in Taiwan over the past 15 years. A retrospective population-based study was conducted using the Chang Gung Research Database. Patients with IK were identified by diagnostic codes for corneal ulcer from 2004 to 2019. Of 7807 included subjects, 45.2% of patients had positive corneal cultures. The proportion of contact lens-related IK declined, while that of IK related to systemic diseases grew. The percentage of isolated gram-positive bacteria surpassed that of gram-negative bacteria in the 15-year period. The prevalence of *Pseudomonas aeruginosa* showed a decreasing trend (*p* = 0.004), whereas coagulase-negative *Staphylococcus* (CNS) and *Propionibacterium* species were increasingly detected (*p* < 0.001). Overall, the trend of antibiotic susceptibility of both gram-positive and gram-negative bacteria did not change throughout the study period. The susceptibility to the test antibiotics maintained over 90% in gram-negative isolates over 15 years. Vancomycin preserved 100% susceptibility to all gram-positive isolates. Since most tested antibiotics exhibited stable susceptibility over decades, this study reinforced that fluoroquinolones and fortified vancomycin continue to be good empiric therapies for treating bacterial keratitis in Taiwan.

## 1. Introduction

Infectious keratitis (IK) is one of the leading causes of ocular morbidity and blindness worldwide [1,2]. Complications related to IK not only cause visual disability but also place a heavy socioeconomic burden on the affected individuals and national health care systems [3,4]. The estimated incidence of IK ranges from 2.5 to 799 cases per 100,000 population per year, varying from geographic locations and study designs [5,6]. In Taiwan, a 14-year population-based study reported an increase in the incidence of IK from 8.3 in 2000 to 20.2 per 100,000 person-years in 2013 [3].

IK can be caused by a wide array of organisms, including bacteria, fungi, protozoa and viruses [7,8]. Polymicrobial infection also accounts for 2–15% of all IK cases [9,10]. Common risk factors for IK encompass contact lens wear, ocular trauma, ocular surface diseases (OSD), preceding ocular surgeries and systemic diseases [11,12]. Corneal scraping culture and stain as the current gold standard for diagnosis and determination of causative pathogens in IK, are time-consuming and may yield no useful results [13]. Timely and appropriate antimicrobial therapy is crucial for the eradication of infection and visual recovery. Accordingly, clinicians usually initiate empiric broad-spectrum treatment, either with fluoroquinolone monotherapy or combining with several fortified antimicrobial regimen, on the basis of clinical findings and regional epidemiological data before the results of diagnostic corneal smears and cultures are available [14,15]. Furthermore, a number of reports have demonstrated the development of bacterial strains resistant to commonly used antimicrobial agents [16,17]. The issue of emerging antimicrobial resistance is rising due to widespread and inappropriate use of broad-spectrum antibiotics [18].

Many published studies have been dedicated to comprehensive global data analyses of microbial keratitis [13,19,20]. However, the microbial spectrum and resistance patterns change greatly over time and vary enormously from region to region [19]. The establishment of updated local data of IK is essential to guide clinical practice. Therefore, the purpose of the present study was to investigate the microbiological and epidemiologic characteristics of IK in the recent decade, and to detect the shifting trends in corneal isolates and their antibiotic susceptibility profiles over time in Taiwan using a population-based database.

## 2. Materials and Methods

### 2.1. Study Design and Data Source

This was a hospital-based retrospective study utilizing the Chang Gung Research Database (CGRD), which is the largest multi-institutional de-identified electronic medical records (EMR) database in Taiwan. Overall, the CGRD includes 21.2% of outpatients and 12.4% inpatients in the Taiwanese population. Owing to high overall and disease-specific coverage, the CGRD provides good access for clinical and scientific studies. The patient-level demographics and data on the health conditions are coded according to the International Classification of Diseases, Ninth Revision, Clinical Modification (ICD-9-CM) codes before 2016 and ICD-10-CM codes after 2016.

### 2.2. Patient Identification

**Figure 1:**
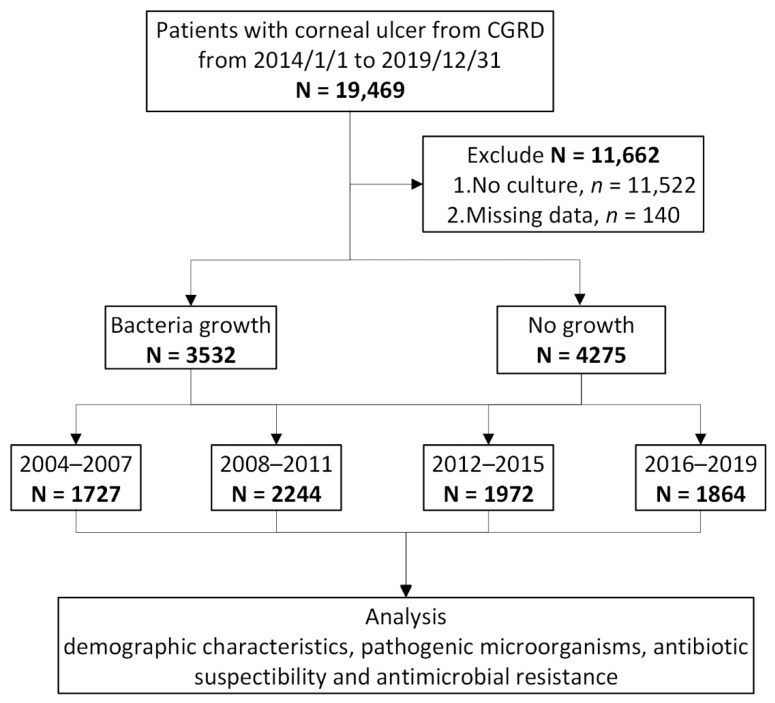
Flowchart of study population selection. CGRD, Chang Gung Research Database.

We enrolled patients with IK from 1 January 2004 to 31 December 2019, using ICD-9-CM and ICD-10 codes: 370.00, 370.01, 370.02, 370.03, 370.04, 370.05, 370.06, 370.55 and H16.00, H16.01, H16.02, H16.03, H16.04, H16.06, H16.07, H16.31. Patients with missing demographic information and without corneal culture data were excluded. The corneal scraping samples were obtained within one week of the index date. All cases were included only once; however, recurrent episodes were not involved in this study (Figure 1).

### 2.3. Determination of Risk Factors

Predisposing factors for IK were determined using the ICD-9-CM and ICD-10 diagnostic codes, procedure codes, and drug prescriptions recorded in the claims data before the diagnosis was recognized (Appendix A). The identifiable risk factors were categorized into five subgroups: contact lens (CL) wear, ocular trauma, recent ocular surgery, OSD and systemic disorders. Without a specific code for contact lens wear, we used diagnostic codes for corneal disorder due to contact lens and contact lens prescription records within 3 months before IK was identified. Ocular trauma was defined by documented presence with a history of traumatic ocular injury within 3 months prior to the diagnosis of IK. Recent ocular surgery performed within 3 months after the diagnosis of IK were considered surgical-related IK. Ocular surface diseases include dry eye, trichiasis, blepharitis, lagophthalmos and exposure keratopathy, neurotrophic keratopathy, corneal transplant status, and chronic topical antiglaucoma agent use for more than 3 months. Systemic disorders with ocular involvement include diabetes mellitus (DM), autoimmune diseases, chronic kidney disease (CKD), atopic dermatitis and human immunodeficiency virus (HIV) infection. These medical comorbidities if appearing at least once in the diagnoses of inpatients or at least three times in the diagnoses of outpatients were included.

### 2.4. Isolates and Antibiotic Susceptibility

Corneal scraping smears and cultures were routinely processed to identify the causative organisms in patients with IK. Noncorneal samples, such as conjunctival swabs and aqueous taps, were excluded in this study. A bacterium isolated from the same patient on more than one occasion was regarded as 1 isolate if having the same spectrum of antibiotic resistance.

According to the Clinical and Laboratory Standard Institute standards for testing antimicrobial susceptibility, in vitro susceptibility was interpreted based on serum standards due to lack of standardized values for topical antibiotics in ocular tissues. Isolates of intermediate susceptibility were categorized as susceptible organisms, since frequent instillation of fortified antibiotics may yield a higher antibiotic concentration in the corneal stroma than that in the serum after systemic administration.

### 2.5. Statistical Analysis

For the purpose of comparison and trend analysis, the study was arbitrarily divided into four time periods stratified according to diagnosis during the 15-year periods: 2004 to 2007, 2008 to 2011, 2012 to 2015, 2016 to 2019. Descriptive statistics such as count and percentage were presented for categorical variables. The Cochran–Armitage trend test and the Mann–Kendall trend test were used to detect the trends. All the analyses were performed using SAS software version 9.4 (SAS Institute, Cary, NC, USA), and the threshold for statistical significance was set at *p* < 0.05.

## 3. Results

### 3.1. Demographics of the Study Population

According to the ICD diagnostic codes, 19,469 patients with IK were identified from the CGRD from 1 January 2004 to 31 December 2019, but patients without complete demographic information or culture data were excluded. A total of 7807 patients, including 3809 men (48.8%) and 3998 women (51.2%), were included in this study. The demographic data are summarized in Table 1. The mean age was 46.9 ± 22.1 years. The patients older than 65 years accounted for 22.6%. From 2004 to 2019, no significant differences in sex and age were demonstrated from between-decade comparisons (*p* = 0.957, *p* = 0.275, respectively). A total of 7807 records of corneal scraping culture from 7807 IK patients with corneal ulcer were reviewed. Among the selected records, 3532 (45.2%) samples were culture positive. The positivity rate was rising from 40% to 52% over the 15-year period (*p* < 0.001).

### 3.2. Predisposing Factors for Infectious Keratitis

Among the patients with recognizable risk factors in the claims data (N = 3268, 41.9%), CL wear accounted for 10%; ocular trauma for 8.8%; recent ocular surgery for 3.2%; OSD for 17.9%; systemic disorders with ocular involvement for 14.2%. From between-decade comparison, an increasing trend in systemic disorder-related IK, particularly in patients with chronic kidney disease, and a decreasing trend in IK associated with prior ocular surgery (*p* < 0.001) were presented (Table 2). Similarly, a significant changing trend in CL-related IK was observed (*p* = 0.001) although the coding rate of “corneal disorder due to contact lens” was low. The rate of CL-related IK rose and reached a peak (12.1%) between 2012 and 2015, but subsequently fell to 9.7%. Moreover, IK associated with exposure keratopathy, lagophthalmos, prior corneal transplantation, topical steroid use, Sjögren syndrome and atopy showed a decline over the 15-year period.

### 3.3. Microbiological Profiles

**Figure 2:**
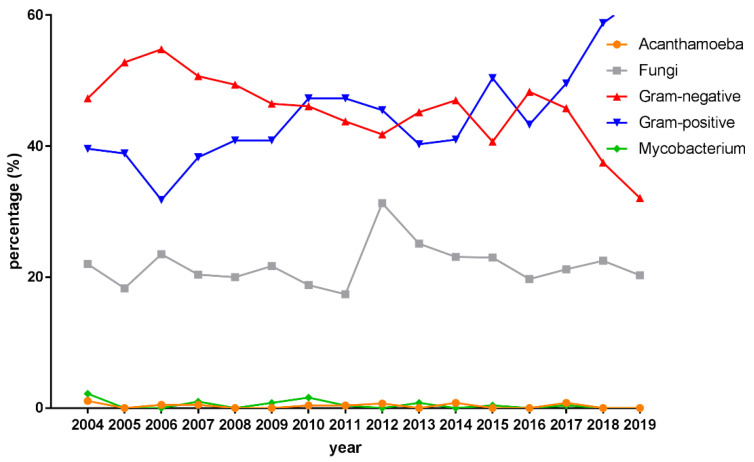
Percentage of microbial isolates from infectious keratitis between 2014 and 2019.

Data from 7807 corneal scraping cultures were available for study. A total of 3990 pathogens were recovered with 26.8% polymicrobial infection in the patients with IK. From 2004 to 2019, gram-positive bacteria accounted for 45.6% isolates, followed by gram-negative bacteria (45.1%), fungal isolates (21.5%), *Mycobacteria* (0.4%) and *Acanthamoeba* (0.3%). Figure 2 illustrates an increasing trend of gram-positive bacteria and a decreasing trend of gram-negative bacteria over the 15-year period. The upward trend of gram-positive bacteria and the downward trend of gram-negative bacteria were both significant (z = 14.77, *p* < 0.001; z = −13.13, *p* < 0.001). In 2012, the proportion of gram-positive bacteria exceeded that of gram-negative bacteria. The percentage of fungal isolates per year ranged from 17.4% to 31.3% for all positive cultures, showing stable over 15 years.

Of all bacterial growths, *Pseudomonas aeruginosa* was the most commonly identified bacterial isolate (N = 1002, 28.4%), followed by *Staphylococcus* species (N = 901, 25.5%), *Propionibacterium* species (N = 264, 7.5%), *Streptococcus* species (N = 192, 5.4%) and *Serratia* species (N = 150, 4.2%) (Table 3). Among *Staphylococcus* species, coagulase-negative *Staphylococcus* (CNS) (N = 631, 17.9% of total bacterial growths and 70.0% of *Staphylococci* isolates) ranked first, followed by *Staphylococcus aureus* (N = 257, 7.3% of total bacterial growths and 28.5% of *Staphylococci* isolates). A notable increase occurred in the percentage of *Staphylococcus* species and *Propionibacterium* species (*p* < 0.001) over the 15-year period, whereas a significant decrease appeared in the percentage of *P. aeruginosa* and *Streptococcus* species (*p* = 0.004, *p* = 0.033, respectively). Interestingly, in the subgroup analysis of *Staphylococci* group, *S. epidermis* was responsible for the upward trend in the overall proportion, whereas *S. aureus* remained stable over the 15-year period.

### 3.4. Antibiotic Susceptibility Patterns

#### 3.4.1. Gram-Negative Isolates

During 2004 to 2019, most isolated gram-negative bacteria were susceptible to β-lactams and fluoroquinolone (piperacillin: 95.4%; ceftazidime: 96%; cefepime: 95.6%; imipenem: 95.7%; meropenem: 95.2%; ciprofloxacin: 95.4%; levofloxacin: 97.1%), followed by aminoglycoside (amikacin: 94.4%; gentamicin: 90.6%), as shown in Table 4. Isolated gram-negative bacteria also continuously exhibited a susceptibility over 90% in all recommended antibiotics in the 15-year period. *P. aeruginosa*, the most common isolate in this study, exhibited more than 95% in vitro susceptibility to all tested antibiotics. Nearly all *P. aeruginosa* isolates were susceptible to piperacillin-tazobactam, ceftazidime, cefepime and amikacin, while 96.1% and 97.8% of *P. aeruginosa* isolates were susceptible to gentamicin and levofloxacin, respectively (Table 5).

#### 3.4.2. Gram-Positive Isolates

As for gram-positive bacteria, more than 95% of isolates were susceptible to glycopeptide, linezolid and fusidic acid (teicoplanin: 99.7%; vancomycin: 99.6%; linezolid: 100%; fusidic acid: 97.3%), while clindamycin covered 75% of the isolates in this study (Table 6). The resistances of oxacillin and erythromycin were observed (63.6%, 877 sensitive in 1379; 52.8%, 970 sensitive in 1837, respectively). Regarding the tested gram-positive isolates, the susceptibility of trimethoprim-sulfamethoxazole (TMP-SMX) increased from 82.8% to 90.2% in the period of 2004–2007 to 2016–2019 (*p* = 0.025). Fluoroquinolones were not on the recommended list of antibiotics for gram-positive bacteria in our microbiologic laboratory. Furthermore, from 2004 to 2019, all isolated *Staphylococcus* species showed 100% susceptibility to vancomycin, whereas the percentage of all *Staphylococci* isolates susceptible to oxacillin was 62.8% (Table 7). The susceptibility of the tested antibiotics against Staphylococcus species had no statistically significant change throughout the four study periods.

#### 3.4.3. Multidrug-Resistant Isolates

During the 15-year period, multidrug-resistant (MDR) bacteria was isolated every year despite representing only a small percentage (N = 144, 4.1%), which was stable without significant change in trend (*p* = 0.05). Methicillin-resistant *S. aureus* (MRSA) accounted for 2.3% of all isolated organisms (5.1% of all Gram-positive organisms; 31.9% of *S. aureus* isolates), followed by vancomycin-resistant *Enterococci* (VRE) (1.4% of all isolates), Carbapenem-resistant *P. aeruginosa* (CRPA) (0.2% of all isolates), extended-spectrum β-lactamase-producing *Escherichia coli* (*E. coli*-ESBL) (0.1% of all isolates) and multidrug-resistant *Acinetobacter baumannii* (MDR-AB) (0.1% of all isolates). For the in vitro susceptibility test, vancomycin retained activity (100%) against MRSA (Table S1).

## 4. Discussion

The spectrum of pathogenic microorganisms and their antimicrobial susceptibility vary with different regions and change over time. Therefore, periodical renewal of the local epidemiology of IK for evidence-based guidance is vital in clinical practice. This study provides updated information about microbiological data and patient demographics of IK in Taiwan.

From our results, the average age (47 years) of the patients with IK was similar to that in previous literature reported worldwide (average age ranges from 42 to 56 years) [20]. Even though no gender predilection was shown, a slight female preponderance was presented through the 15-year period. This finding is consistent with prior domestic studies indicating that IK occurs more commonly in females in Taiwan [3,21]. The data of the Asia Cornea Society Infectious Keratitis Study also suggested that IK was associated with a female predominance in Taiwan, Japan and Singapore [8].

Regarding the risk factors for IK, OSD represented the relatively main identified predisposing factor in this study, which was different from other studies [3,8,10,11,20,22]. Ocular surface diseases have become a popular issue in recent decades. Dysregulation of the ocular surface may lead to ocular surface inflammation and damage of the corneal epithelium, consequently increasing the risk of IK [23]. A 5-year Australian study revealed that IK in patients with a history of ocular surface diseases were more likely to have longer recovery time and less favorable outcomes [10].

Moreover, IK associated with systemic disorders showed a significantly increasing trend, while a decreasing trend of IK presented in patients with preceding ocular surgery. In subgroup analysis, DM and CKD were shown as vital elements contributing to the upward trend. In accordance with our findings, prior studies have reported DM as a risk factor for microbial keratitis [3,24,25]. Existing literature explained that hyperglycemia facilitates microbial growth and inhibits host immune response to infection [26,27]. DM can alter corneal nerve plexuses and affect ocular surface homeostasis, thereby increasing the risk of IK [28,29]. In terms of CKD, a Taiwanese study demonstrated that end-stage renal disease (ESRD) increased 1.17 times of risk to develop corneal ulcer, particularly in patients with DM [30]. The author assumed that compromised ocular surface and quality of tear film may predispose patients to IK [30]. Weng et al. showed that ESRD increases the risk of band keratopathy, which disrupts the regularity of the ocular surface and then prompt colonization of organisms and tissue invasion [31]. Furthermore, several reports suggested that the activation of toll-like receptors expressed on the corneal epithelium may be involved in the pathogenesis of corneal infection by exacerbating various ocular surface inflammation [32].

CL wear had been shown to be the most common risk factor for microbial keratitis in the United State, Europe and Australia [33,34,35]. Previous domestic surveys also indicated that CL wear was the most common documented risk factor of IK with incidence around 31–44% [12,21,36]. Since our results were analyzed based on the ICD diagnostic coding at clinic, we could not accurately identify the exact number of “corneal disorders due to CL use” in the CGRD. In general, the history of CL use was routinely recorded as text rather than code in the medical charts. Intriguingly, in line with previous literature, the number of CL-related IK declined in the present study [21,37]. Liu et al., postulated that the reduction of CL-related IK reflected the rising popularity of daily-disposable lenses or routine application of topical fluoroquinolone eye drops as initial treatment for CL-related ocular disorders [21]. By contrast, in the 2019Think Tank, the American Academy of Optometry (AAO) stated that the rate of CL-related infections had not decreased over three decades despite technology innovation, but they acknowledged that extended wear, such as overnight orthokeratology lenses, increases the risk of IK, whereas daily disposable modalities may minimize the risk of severe corneal infections [38].

The average positive culture rate of the present study was comparable with that in other reports, ranging from 40–68%, and the positive rate increased through the 15-year period [16,19,21,36]. The relatively low rate with a slightly upward trend may be due to the increase of early referral before application of antibiotics [16,21].

Among the isolated microorganisms, the common bacterial isolates were *Pseudomonas* species, *Staphylococcus* species, *Propionibacterium* species, *Streptococcus* species and *Serratia* species, which was consistent with other published reports from Taiwan [21,39]. Interestingly, our findings revealed that the percentage of gram-positive bacteria significantly increased, surpassing that of gram-negative isolates for the first time by 2010. Although *P. aeruginosa* remained the most commonly isolated pathogen during the study period, the percentage declined significantly. The similar phenomenon of shifting trends of isolates was also presented in the studies from the UK and Iran, but not shown in the previous domestic reports [21,36,39,40,41]. *Pseudomonas* species are responsible for CL- and trauma-related IK in most settings [8]. The decline in the percentage of *Pseudomonas* species may be attributable to the widespread use of fluoroquinolones and advanced hygiene concepts in contact lens use [19]. The reduction in the rate of corneal scrapes in CL-related infections and early recovery of IK might be another reason for the decreasing percentage of Pseudomonas isolates [37]. On the other hand, coagulase-negative *Staphylococcus* (CNS), particularly *S. epidermis*, was the main culprit pathogen of staphylococcal keratitis, the rise of which is one of the leading causes of an increase in the percentage of gram-positive isolates. Likewise, previous studies of bacterial keratitis from India, the UK, New Zealand and Canada, have demonstrated that CNS was the most common causative organism in IK, ranging from 24.8% to 40.8% of the isolates [16,42,43,44]. In a ten-year analysis of microbial keratitis conducted in the UK, Ting et al. consistently observed an increasing trend in Gram-positive organisms, particularly CNS, and a decreasing trend in Gram-negative organisms, particularly *Pseudomonas* [40]. In contrast, Tam et al. found a decreasing trend in the number of isolates in gram-positive microorganisms over the past 16 years [45].

With respect to the virulence, CNS has always been considered a group of common ocular commensal that opportunistically causes endophthalmitis, keratitis, and blepharoconjunctivitis [46]. However, the identification of CNS as a pathogen varies among laboratories. The increasing trend in the percentage of CNS may reflect both the inherent nature of geographical prevalence in Taiwan and the likelihood of contamination of cornea [39]. We hypothesized that some systemic disorders, such as DM and CKD, may be associated with the increase of CNS isolates in Taiwan. These systemic diseases involving eyes often develop OSD, among which the OSD-related IK was caused by CNS and *S. aureus* [33,47]. Once normal flora of the ocular surface and a contaminant were considered during scraping, *Propionibacterium* species implicated in IK showing an increasing trend of isolation has been drawn attention in recent evidence [39,48].

Our study indicated that gram-negative bacteria preserve better susceptibility to the tested antibiotics compared with gram-positive bacteria, supporting evidence from previous reports [16,36,39,44,45,49]. The susceptibility of gram-negative bacteria to tested antibiotics seemed stable and maintained 90% over 15 years [7,16,19,39,45]. Most gram-negative isolates were susceptible to fluoroquinolone (96.3%) and cephalosporin (95.8%), followed by aminoglycoside (92.5%) in the present study. Interestingly, we found a significant increase of susceptibility to amikacin against gram-negative bacteria except *P. aeruginosa*. Nevertheless, topical fluoroquinolones have substantially replaced combined fortified aminoglycosides and cephalosporins as an empiric treatment for bacterial keratitis, regarding its low ocular toxicity and commercial availability [16,17]. In our institution, our empiric therapy for IK has been changed to levofloxacin in recent years.

Fluoroquinolones are currently widespread used as empiric therapy in bacterial keratitis due to the broad coverage of spectrum, low toxicity and good absorption to the ocular surface [14,15]. However, emerging resistance to the antibiotics has been increasingly reported worldwide over the last two decades [17,49,50]. Nevertheless, susceptibility to levofloxacin in gram-negative bacteria, especially *P. aeruginosa*, remained high and unchanged, ranging from 96% to 98%, during all study periods in Taiwan. This discrepancy could be attributed to variations between different geographical locations. However, the lack of data hinders further analyses regarding the susceptibility test against fluoroquinolones through the study period.

In gram-positive bacteria, most isolates were resistant to macrolide (52.8% sensitivity of all gram-positive isolates), similar to the global data, 57% [19]. Beyond our expectation, the susceptibility rate to TMP-SMX for gram-positive bacteria increased significantly from 82.1% to 90.2%. The average susceptibility to TMP-SMX was 85.8%, similar to that in reports from Toronto, Canada [45]. Meanwhile, a slight decline trend of antimicrobial resistance against oxacillin and clindamycin, regarded as antibiotic selective pressure, was observed, showing consistency with a previous domestic report [39].

The present study examined that nearly 40% of all *Staphylococci* were resistant to oxacillin with 31.9% being MRSA, whereas the study by Hsiao et al., showed 40% of CNS and *S. aureus* isolates were oxacillin-resistant in a 10-year single-center study [39]. Our results presented no significant change in trend regarding the susceptibility to oxacillin for *S. aureus* in Taiwan. In the Toronto study, Lichtinger et al. found that 29.1% of all gram-positive bacteria were methicillin-resistant isolates, while 43.1% of CNS and 1.3% of *S. aureus* isolates were oxacillin-resistant [42].

Multiple drug resistance has had a global impact on public health in the field of ophthalmology [51,52]. The development of antimicrobial resistance is multifactorial, including injudicious use of antimicrobial agents, genetic mutational resistance and horizontal gene transfer of microorganisms per se [53]. Several recent studies have demonstrated the emergence of antibiotic resistance in ocular infections [16,19,39,42,45,49,50]. A certain proportion of antimicrobial-resistant bacteria among the isolated strains appeared annually in our study. Reassuringly, the percentage of MDR bacteria, such as MRSA, did not increase over time. All gram-positive bacteria, including methicillin-resistant isolates, were susceptible to vancomycin (100% sensitivity), the last resort for MRSA. In another domestic study in Taiwan, fluoroquinolones were effective against *S. aureus* [39]. Therefore, we may consider fluoroquinolones as initial empiric treatment for IK and combination regimen with fortified vancomycin for the severe IK cases in Taiwan. Nevertheless, we should appropriately use these second-line antibiotics to prevent the emergence of antimicrobial resistance.

Our study presents several strengths and limitations. To our knowledge, this is the first study to investigate the demographic and microbiological characteristics of IK in Taiwan with the broadest range of study period. Furthermore, the database research contains a large number of cases from multiple hospitals, at least one third of medical coverage of Taiwan; therefore, the findings of this study could represent real-world evidence. However, the retrospective nature restricted detailed review of clinical information such as initial appearance, prior antibiotic use and contact lens wear, which are unavailable in the claims database. CL-related IK was difficult to directly identify in our database since the coding rate of “corneal disorder due to contact lens” was low. The similar problem happened to other predisposing factors, causing underestimate of the incidence. Regarding limitations on the microbiological study, in vitro antibiotic susceptibility interpreted based on serum standards could not provide accurate assessment of antibiotic resistance in ocular strains. To identify whether CNS and *Propionibacterium* isolates are either pathogens or commensal species from the ocular surface remains a challenge. Therefore, further studies are required to comprehensively clarify species correlations with ocular infections. As with other epidemiological studies, our findings should not be generalized to other geographic regions or populations.

## 5. Conclusions

The ratio of the isolated gram-positive bacteria and gram-negative bacteria presented a significant cross. Simultaneously, we found an increasing trend in the percentage of IK associated with systemic diseases, particularly CKD and DM, whereas the proportion of CL-related IK declines. As positive culture rate rises, CNS keratitis and *Propionibacterium* keratitis are regarded as potential ocular surface infections that warrant more attention. However, *P. aeruginosa* remains the most frequently isolated bacteria responsible for IK in Taiwan. Since no significant change in antibiotic susceptibility and the percentage of MDR strains were noticed, this study highlights that fluoroquinolones and fortified vancomycin continue to be good empiric therapies for treating bacterial keratitis in Taiwan.

## Figures and Tables

**Table 1:** Demographic characteristics of patients with infectious keratitis between 2004 to 2019.

	2004–2007 N = 1727	2008–2011 N = 2244	2012–2015 N = 1972	2016–2019 N = 1864	
	*n* (%)	*n* (%)	*n* (%)	*n* (%)	*p*-Value *
Sex					0.957
Male	810 (46.9%)	1146 (51.1%)	955 (48.4%)	898 (48.2%)	
Female	917 (53.1%)	1098 (48.9%)	1017 (51.6%)	966 (51.8%)	
Age (years)					0.275
≤65	1300 (75.3%)	1698 (75.7%)	1487 (75.4%)	1375 (73.8%)	
>65	427 (24.7%)	546 (24.3%)	485 (24.6%)	489 (26.2%)	
Mean ± SD	45.5 ± 22.1	46.0 ± 22.1	47.5 ± 21.7	48.5 ± 22.5	
Culture rate					<0.001
Number of bacterial growth	689 (40%)	992 (44%)	874 (44%)	977 (52%)	
No growth	1038 (60%)	1252 (56%)	1098 (56%)	887 (48%)	

* Cochran-Armitage trend test.

**Table 2:** Distribution of identifiable predisposing factors for infectious keratitis.

	2004–2007 N = 1727	2008–2011 N = 2244	2012–2015 N = 1972	2016–2019 N = 1864	
Variables	*n* (%)	*n* (%)	*n* (%)	*n* (%)	*p*-Value *
Contact lens wear	105 (6.1%)	254 (11.3%)	238 (12.1%)	181 (9.7%)	0.001
Ocular trauma	138 (8.0%)	235 (10.5%)	174 (8.8%)	141 (7.6%)	0.236
Recent ocular surgery	82 (4.8%)	83 (3.7%)	42 (2.1%)	43 (2.3%)	<0.001
Ocular surface disease	312 (18.1%)	418 (18.6%)	358 (18.2%)	313 (16.8%)	0.264
Dry eye	104 (6.0%)	176 (7.8%)	155 (7.9%)	147 (7.9%)	0.052
Trichiasis	26 (1.5%)	37 (1.7%)	30 (1.5%)	16 (0.9%)	0.083
Blepharitis	112 (6.5%)	176 (7.8%)	139 (7.1%)	108 (5.8%)	0.226
Exposure keratopathy, or lagophthalmos	21 (1.2%)	29 (1.3%)	24 (1.2%)	7 (0.4%)	0.012
Neurotrophic keratopathy	3 (0.2%)	7 (0.3%)	10 (0.5%)	6 (0.3%)	0.295
Corneal transplantation status	93 (5.4%)	72 (3.2%)	58 (2.9%)	52 (2.8%)	<0.001
Topical antiglaucoma agents	60 (3.5%)	57 (2.5%)	57 (2.9%)	65 (3.5%)	0.744
Topical steroid	46 (2.7%)	47 (2.1%)	26 (1.3%)	31 (1.7%)	0.009
Systemic disorder	216 (12.5%)	301 (13.4%)	292 (14.8%)	297 (15.9%)	0.001
Diabetes mellitus	152 (8.8%)	226 (10.1%)	213 (10.8%)	197 (10.6%)	0.061
Non-Sjörgen autoimmune dz (RA, SLE, AS, other CTDs…)	51 (3.0%)	63 (2.8%)	47 (2.4%)	70 (3.8%)	0.255
Sjögren syndrome	44 (2.6%)	40 (1.8%)	25 (1.3%)	27 (1.5%)	0.006
Atopy	10 (0.6%)	12 (0.5%)	16 (0.8%)	22 (1.2%)	0.020
Chronic kidney disease	27 (1.6%)	53 (2.4%)	54 (2.7%)	67 (3.6%)	<0.001
HIV infection	1 (0.1%)	3 (0.1%)	7 (0.4%)	4 (0.2%)	0.128

RA, rheumatoid arthritis; SLE, systemic lupus erythematosus; AS, ankylosing spondylitis; CTD, connective tissue disease; HIV, human immunodeficiency virus. * Cochran-Armitage trend test.

**Table 3:** Most common bacterial isolates from corneal scrapes between 2004–2007, 2008–2011, 2012–2015 and 2016–2019.

	2004–2007 N = 689	2008–2011 N = 992	2012–2015 N = 874	2016–2019 N = 977	Total N = 3532	
Bacteria	*n* (%)	*n* (%)	*n* (%)	*n* (%)	*n* (%)	*p*-Value *
*Staphylococcus* spp.	140 (20.3)	248 (25)	193 (22.1)	320 (32.8)	901 (25.5)	<0.001
*S. aureus*	53 (7.7)	68 (6.9)	55 (6.3)	81 (8.3)	257 (7.3)	0.619
*S. epidermis*	26 (3.8)	50 (5.0)	67 (7.7)	169 (17.3)	312 (8.8)	<0.001
Other CNS	63 (9.1)	138 (13.9)	67 (7.7)	51 (5.2)	319 (9)	<0.001
*Streptococcus* spp.	55 (8)	49 (4.9)	36 (4.1)	52 (5.3)	192 (5.4)	0.033
*S. pneumoniae*	34 (4.9)	27 (2.7)	13 (1.5)	16 (1.6)	90 (2.5)	<0.001
*Propionebacterium* spp.	14 (2.0)	90 (9.1)	71 (8.1)	89 (9.1)	264 (7.5)	<0.001
*Pseudomonas* spp.	229 (33.2)	283 (28.5)	280 (32)	254 (26)	1046 (29.6)	0.012
*P. aureuginosa*	225 (32.7)	277 (27.9)	252 (28.8)	248 (25.4)	1002 (28.4)	0.004
*Serratia* spp.	43 (6.2)	49 (4.9)	29 (3.3)	29 (3)	150 (4.3)	<0.001

CNS, coagulase-negative staphylococcus. * Cochran-Armitage trend test.

**Table 4:** Summary of in vitro antibiotic susceptibility of gram-negative isolates between 2004–2007, 2008–2011, 2012–2015 and 2016–2019.

	2004–2007	2008–2011	2012–2015	2016–2019	Total	
Antibiotics	*n* (%)	*n* (%)	*n* (%)	*n* (%)	*n* (%)	*p*-Value *
Piperacillin	298 (94.6)	346 (94.8)	302 (97.0)	69 (94.2)	1015 (95.4)	0.365
Ceftazidime	324 (96.3)	417 (94.7)	355 (96.6)	349 (96.6)	1445 (96.0)	0.517
Cefepime	260 (95.8)	329 (93.9)	311 (96.1)	278 (96.8)	1178 (95.6)	0.305
Imipenem	313 (97.4)	338 (95.3)	311 (94.9)	278 (95.0)	1240 (95.7)	0.132
Meropenem	220 (95.5)	332 (94.0)	309 (95.8)	270 (95.9)	1131 (95.2)	0.506
Gemamicin	323 (90.1)	415 (88.9)	355 (92.1)	338 (91.7)	1431 (90.6)	0.231
Amikacin	325 (92.0)	415 (94.0)	355 (94.9)	338 (96.5)	1433 (94.4)	0.012
Ciprofloxacin	326 (95.1)	414 (94.2)	357 (96.6)	338 (95.6)	1435 (95.4)	0.449
Levofloxacin	105 (96.2)	50 (98.0)	235 (97.5)	338 (97.0)	728 (97.1)	0.792

* Cochran-Armitage trend test.

**Table 5:** Susceptibility of *Pseudomonas aeruginosa* isolates to the tested antibiotics.

	2004–2007	2008–2011	2012–2015	2016–2019	Total	
Antibiotics	*n* (%)	*n* (%)	*n* (%)	*n* (%)	*n* (%)	*p*-Value *
Piperacillin/tazobacatm	126 (98.4)	268 (98.1)	245 (98.4)	234 (99.6)	873 (98.7)	0.247
Ceftazidime	214 (99.5)	268 (99.6)	246 (99.6)	235 (99.6)	963 (99.6)	0.969
Cefepime	214 (99.5)	262 (99.2)	246 (99.2)	235 (99.6)	957 (99.4)	0.956
Amikacin	214 (99.1)	268 (98.9)	246 (99.6)	235 (100.0)	963 (99.4)	0.120
Gentamycin	214 (95.8)	268 (94.8)	246 (97.2)	235 (96.6)	963 (96.1)	0.377
Ciprofloxacin	214 (97.7)	268 (97.8)	246 (98.8)	235 (97.9)	963 (98.0)	0.685
Levofloxacin	80 (96.3)	N/A	142 (98.6)	235 (97.9)	457 (97.8)	0.438

N/A = not applicable. * Cochran-Armitage trend test.

**Table 6:** Summary of in vitro antibiotic susceptibility of gram-positive isolates between 2004–2007, 2008–2011, 2012–2015 and 2016–2019.

	2004–2007	2008–2011	2012–2015	2016–2019	Total	
Antibiotics	*n* (%)	*n* (%)	*n* (%)	*n* (%)	*n* (%)	*p*-Value *
Penicillin	N/A	10 (90.0)	11 (90.9)	15 (73.3)	36 (83.3)	0.238
Oxacillin	133 (57.9)	229 (63.8)	190 (60.0)	325 (68.0)	877 (63.6)	0.062
Teicoplanin	170 (100.0)	257 (99.6)	222 (99.6)	361 (99.7)	1010 (99.7)	0.715
Vancomycin	168 (99.4)	278 (100.0)	232 (100.0)	374 (99.2)	1052 (99.6)	0.383
Linezolid	10 (100.0)	40 (100.0)	39 (100.0)	63 (100.0)	152 (100.0)	NA
Erythromycin	158 (51.9)	248 (52.0)	214 (52.8)	350 (53.7)	970 (52.8)	0.643
Clindamycin	172 (71.5)	335 (75.5)	286 (74.8)	434 (76.0)	1227 (75.0)	0.366
TMP-SMX	145 (82.8)	233 (84.6)	190 (82.1)	325 (90.2)	893 (85.8)	0.025
Fusidic acid	NA	51 (96.1)	51 (98.0)	81 (97.5)	183 (97.3)	0.656

TMP-SMX = trimethoprim-sulfamethoxazole; N/A = not applicable. * Cochran-Armitage trend test.

**Table 7:** Susceptibility of *Staphylococci* isolates to the tested antibiotics.

	2004–2007	2008–2011	2012–2015	2016–2019	Total	
Antibiotics	*n* (%)	*n* (%)	*n* (%)	*n* (%)	*n* (%)	*p*-Value *
Penicillin	129 (14.0)	229 (20.1)	187 (13.4)	312 (22.4)	857 (18.6)	0.099
Oxacillin	133 (57.9)	229 (63.8)	187 (59.4)	312 (66.4)	861 (62.8)	0.157
Vancomycin	126 (100.0)	229 (100.0)	187 (100.0)	311 (100.0)	853 (100.0)	NA
Erythromycin	133 (51.9)	229 (50.7)	187 (52.9)	312 (55.1)	861 (53.0)	0.353
Clindamycin	133 (69.9)	229 (68.1)	187 (67.9)	312 (72.1)	861 (69.8)	0.467
TMP-SMX	133 (85.0)	229 (84.7)	187 (81.8)	312 (90.1)	861 (86.1)	0.097

TMP-SMX = trimethoprim-sulfamethoxazole. * Cochran-Armitage trend test.

## Data Availability

The data presented in this study are available upon request from the corresponding author. The data are not publicly available due to ethical restrictions.

## Supplementary Materials

The following supporting information can be downloaded at: https://www.mdpi.com/article/10.3390/diagnostics12092095/s1, Table S1: Susceptibility of methicillin-resistant *Staphylococcus aureus* (MRSA) isolates to the tested antibiotics.

## Appendix A

**Table A1:** International Classification of Diseases, 9th Revision, Clinical Modification codes (ICD-9-CM code) and ICD-10 for various conditions.

Conditions	ICD-9 Codes	ICD-10 Codes
Contact lens use (= Corneal disorder due to contact lens)	71.82, 367.0, 367.1, 367.2, 367.9	H18.82, H52.0X, H52.1, H52.2, H52.7
Ocular trauma	18, 918.0, 918.1, 918.2, 918.9	S00.2, S00.01, S00.02, S05.00, S05.01, S05.9
Dry eye	375.15, 370.33, 710.2	H04.12, H16.22, M35.00, M35.01, M35.09
Trichiasis	374.05	H02.05
Blepharitis	372.20, 372.21, 372.22, 373.00, 373.01, 373.02, 373.31, 373.32, 373.33, 373.34	H10.50, H10.52, H10.53, H01
Exposure keratopathy, or lagophthalmos	370.34, 374.2	H16.21, H02.2
Neurotrophic keratopathy	370.35	H16.23
Corneal transplant status	V42.5	Z94.7
Diabetes mellitus	250	E11, E10, E09, E13, E08
Sjögren syndrome	710.2	M35.0
Autoimmune diseases (Non-Sjörgen)	701.0, 710.1, 710.3, 710.4, 710.5, 710.8, 710.9, 714.0, 714.1, 714.2, 714.30, 714.31, 714.32, 714.33, 720, 725	L90.0, L94.0, L94.1, L94.3, M32, M34, M35.1-9, M33, M05, M06, M08, M45, M46, M48, M49
Atopic dermatitis	691.8, 372.05	L20.8, L20.9, H10.1
Chronic kidney disease	585	N18.4-6, N18.9
Human immunodeficiency virus infection	V08, 795.71, 042	Z21, Z22.6, R75, B20, B21, B22, B23, B24
