# Supplementary material for: Trends in Infectious Keratitis in Taiwan: An Update on Predisposing Factors, Microbiological and Antibiotic Susceptibility Patterns
Source: Diagnostics (Basel). 2022 Aug 29;12(9):2095. doi: 10.3390/diagnostics12092095 (PMC9497811; doi:10.3390/diagnostics12092095)
Supplement: Supplementary file 1 [file diagnostics-12-02095-s001.zip › diagnostics-1821417-supplementary.pdf]

Table S1. Susceptibility of methicillin-resistant *Staphylococcus aureus* (MRSA) isolates to the tested antibiotics.

	2004-2007	2008-2011	2012-2015	2016-2019	Total	
Antibiotics	n (%)	n (%)	n (%)	n (%)	n (%)	P value*
Clindamycin	19 (5.3)	25 (16.0)	19 (21.1)	19 (36.8)	82 (19.5)	0.015
Erythromycin	19 (0.0)	25 (4.0)	19 (21.1)	19 (31.6)	82 (13.4)	0.001
Fusidic acid	N/A	22 (90.9)	18 (100.0)	19 (100.0)	59 (96.6)	0.235
Linezolid	N/A	14 (100.0)	14 (100.0)	14 (100.0)	42 (100.0)	
Smx-tmp	19 (79.0)	25 (76.0)	19 (84.2)	19 (100.0)	82 (100.0)	0.053
Teicoplanin	19 (100.0)	25 (100.0)	19 (100.0)	19 (100.0)	82 (100.0)	
Tigecycline	N/A	25 (100.0)	18 (100.0)	19 (100.0)	62 (100.0)	
Vancomycin	16 (100.0)	25 (100.0)	19 (100.0)	19 (100.0)	79 (100.0)	

* Cochran-Armitage trend test
